# Diversity and dynamics of *Archaea* in an activated sludge wastewater treatment plant

**DOI:** 10.1186/1471-2180-12-140

**Published:** 2012-07-11

**Authors:** Nils Johan Fredriksson, Malte Hermansson, Britt-Marie Wilén

**Affiliations:** 1 Department of Civil and Environmental Engineering, Water Environment Technology, Chalmers University of Technology, Gothenburg, Sweden; 2 Department of Chemistry and Molecular Biology, Microbiology, University of Gothenburg, Gothenburg, Sweden

## Abstract

**Background:**

The activated sludge process is one of the most widely used methods for treatment of wastewater and the microbial community composition in the sludge is important for the process operation. While the bacterial communities have been characterized in various activated sludge systems little is known about archaeal communities in activated sludge. The diversity and dynamics of the *Archaea* community in a full-scale activated sludge wastewater treatment plant were investigated by fluorescence in situ hybridization, terminal restriction fragment length polymorphism analysis and cloning and sequencing of 16S rRNA genes.

**Results:**

The *Archaea* community was dominated by *Methanosaeta*-like species. During a 15 month period major changes in the community composition were only observed twice despite seasonal variations in environmental and operating conditions. Water temperature appeared to be the process parameter that affected the community composition the most. Several terminal restriction fragments also showed strong correlations with sludge properties and effluent water properties. The *Archaea* were estimated to make up 1.6% of total cell numbers in the activated sludge and were present both as single cells and colonies of varying sizes.

**Conclusions:**

The results presented here show that *Archaea* can constitute a constant and integral part of the activated sludge and that it can therefore be useful to include *Archaea* in future studies of microbial communities in activated sludge.

## Background

The activated sludge process is one of the most widely used methods for treatment of wastewater. An important factor for the operation of activated sludge wastewater treatment plants (WWTPs) is the solid-liquid separation in which flocculation and settling are important processes [1]. Poor flocculation and settling of the activated sludge lead to poor effluent quality and can cause environmental problems in the receiving waters. The sludge characteristics depend on the microbial community composition [2-4], the microbial activity [5] and the properties of the extra-cellular polymeric substances in the flocs [6,7]. The bacterial community has been characterized in a number of activated sludge systems [8,9] but very little is known about archaeal communities in sludge. The presence of *Archaea* in activated sludge has been shown by fluorescence in situ hybridization (FISH), e.g. [10]. Methanogens [11,12] and putative ammonia-oxidizing *Archaea* (AOA) [13-15] have been detected by amplification of 16S rRNA and archaeal ammonia monooxygenase subunit A genes. Although present, *Archaea* seem to be of minor importance for both nitrogen and carbon removal [11,16]. However, it is still possible that the *Archaea* have other functions or affect the properties of the activated sludge. Addition of methanogens to the sludge in intermittently aerated bioreactors increased the rates of specific oxygen uptake, denitrification and nitrification suggesting a symbiotic relationship with *Bacteria *[17]. The composition of the methanogenic community in anaerobic sludge has been shown to be crucial for the structure and integrity of granules [18-20] and if methanogens are present in activated sludge they may contribute to the floc structure.

This study had three aims. The first was to describe the *Archaea* community in the activated sludge of a full-scale WWTP by cloning and sequencing of 16S rRNA genes. Although there are many studies where activated sludge samples have been screened for the presence of AOA (e.g. [13-15]), to our knowledge there are only two published studies on the diversity of *Archaea* in activated sludge from a full-scale WWTP [11,12]. One of the studies investigated two small WWTPs [11] and the other a seawater-processing WWTP [12]. The Rya WWTP is a large WWTP treating municipal and industrial wastewater, thus different from the WWTPs in those two studies. Since little is known about *Archaea* in WWTPs and, importantly, sequence coverage for *Archaea* from WWTPs is still modest, the 16S rRNA sequences we obtained here would indicate if published FISH probes were relevant. If so, the second aim was to quantify the *Archaea* by confocal microscopy and FISH and to determine their localization in the flocs. The third aim was to follow the dynamics of the *Archaea* community for a longer period of time using terminal restriction fragment length polymorphism (T-RFLP) analysis. For the third aim, the samples that were used were collected for previous studies of the dynamics of the floc composition and flocculation and settling properties of the activated sludge at the Rya WWTP [21,22]. Analyzing the *Archaea* community in the same samples could give an indication of possible causes or effects of *Archaea* community changes.

## Results

### Observed and estimated richness of the *Archaea* community in the activated sludge

**Figure 1:**
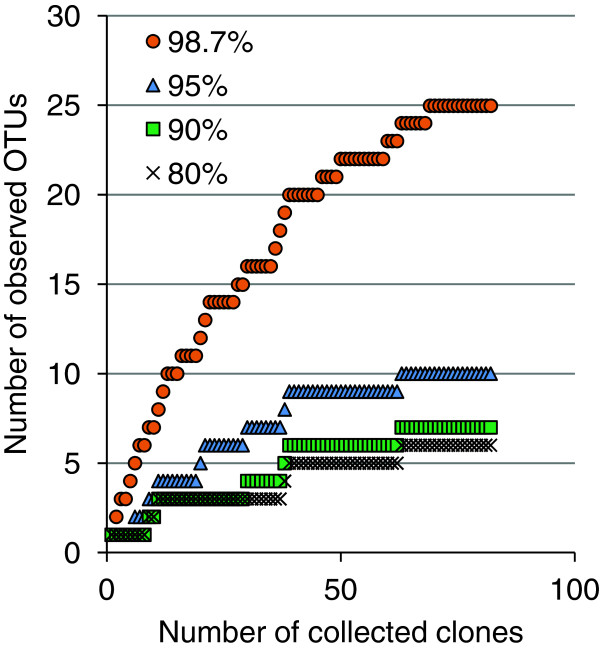
**Accumulation curves of archaeal 16S rRNA gene sequences.** 82 archaeal 16S rRNA gene sequences were assigned to OTUs based on similarity thresholds representing the division in phylum (80%), family/class (90%), genus (95%) and species (98.7%) levels [23,24]. The accumulation curves show the rate of discovery of new OTUs at the different sequence similarity thresholds.

A 16S rRNA gene clone library was constructed from a sample of activated sludge collected at the aeration tank of the Rya WWTP at a time of normal operating conditions. There were no atypical process parameter values or extreme events prior to sample collection. However, the F/M-ratio was higher at the time of the clone library sample collection (May 2007) compared with the times when samples were collected for FISH (December 2007) and T-RFLP analyses (May 2003 - August 2004) (Table 1). Cloning and sequencing generated 82 archaeal 16S rRNA gene sequences of lengths between 756 and 862 bases. Based on DNA similarity the sequences were assigned to operational taxonomic units (OTUs). The sequences were assigned to OTUs corresponding to 25 species of 10 genera, 7 families/classes and 6 different phyla. The *Archaea* community richness was estimated to be at least 43 species of 19 different genera. Thus, the clone library covered at most 58% of the species and 53% of the genera present in the activated sludge. Accumulation curves (Figure 1) also illustrate that the clone library does not fully cover the *Archaea* community.

**Table 1:** Comparison of WWTP parameters at the different sample collection times ^a
^

**Parameter**^**b, c, d**^	**May 03 - Aug 04**^**e**^	**May 07 **^**f**^	**Dec 07**^**g**^	**Comment**
**Temp**^**b**^	15 ± 3	15 ± 1	11 ± 1 **	
**SRT**^**b**^	3 ± 1	3 ± 0	2 ± 0	
**F/M**^**b**^	0.008 ± 0.002	0.014 ± 0.004 **	0.008 ± 0.002	Max value in May 2007
**COD**^**b**^	1058 ± 240	999 ± 194	1068 ±97±	
**NO23-N**^**b**^	48 ± 8	46 ± 9	42 ± 22	Min and max values in Dec 07
**SSVI**^**c**^	80 ± 15	54	79	
**Effluent NSS**^**c**^	23 ± 17	26	31	

^a^ The three periods were compared using the Kruskal-Wallis test. A statistically significant difference, p(same) < 0.05, is marked with asterisks (**). ^b^ Average values (± standard deviation) from all sample dates and the six days preceding the sample dates. ^c^ Data only from sample dates, not including the six preceding days. ^d^ The parameters are water temperature (Temp, °C), solids retention time (SRT, days), food to mass ratio (F/M, g/kg*s), COD going into the activated sludge tanks (COD, g/s), nitrite/nitrate levels going in to the activated sludge tanks (NO23-N, g/s), standardized sludge volume index (SSVI, ml/g) and effluent non-settleable solids (Effluent NSS, mg/l). ^e^ Samples collected during this period were used for T-RFLP analysis. ^f^ A sample collected during this period was used for T-RFLP and clone library analysis. ^g^ A sample collected during this period was used for FISH analysis.

### Evenness and functional organization

**Figure 2:**
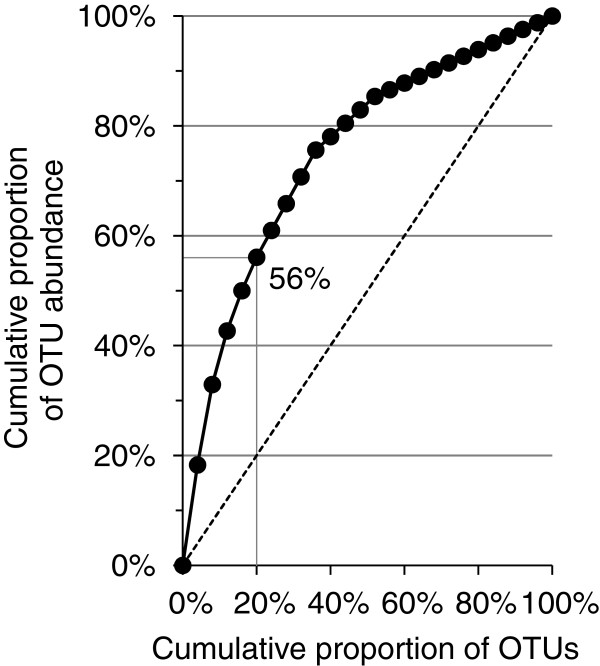
**Pareto-Lorenz evenness curve.** 82 archaeal 16S rRNA gene sequences were divided in 25 OTUs based on a sequence similarity threshold of 98.7% and the OTUs were ranked from high to low, based on their abundance. The Pareto-Lorenz evenness curve is the plot of the cumulative proportion of OTU abundances (y-axis) against the cumulative proportion of OTUs (x-axis). The Fo index, i.e. the combined relative abundance of 20% of the OTUs, is shown. The dotted straight line is the Pareto-Lorenz curve of a community with perfect evenness.

**Figure 3:**
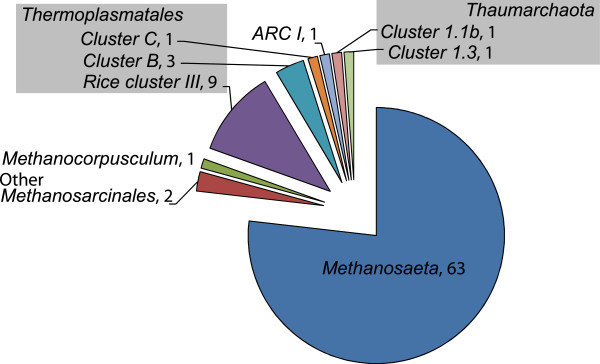
**Community composition.** The 82 16S rRNA gene sequences were classified according to the phylogenetic tree analysis. The number of sequences within each group is given.

Figure 2 shows a Pareto-Lorenz evenness curve of the *Archaea* community based on the relative abundances of the 25 OTUs obtained by applying a 98.7% sequence similarity threshold. The functional organization (Fo) index, the combined relative abundance of 20% of the OTUs, is 56%, meaning that more than half of the observed sequences belong to only five of the observed OTUs. A high Fo index is an indication of a specialized community since it means that a big part of the population belongs to a small number of OTUs and performs a small number of ecological functions. In a completely even community all OTUs would have the same number of individuals and it would be possible for a large number of different functions to be equally abundant. In the clone library, the five most abundant OTUs, which include 56% of the sequences, all belong to *Methanosaeta* and presumably are all methanogens. Furthermore, the composition of the clone library indicates that the community includes a small number of ecological functions since 13 of 25 OTUs, including 77% of the sequences, were identified as *Methanosaeta* (Figure 3).

### Comparison with available sequences in GenBank and SILVA

Searches in GenBank using BLAST [25] and in the SILVA rRNA database [26] found sequences with a sequence similarity of 98.7% or higher for 22 of 25 OTUs, including 78 of the 82 sequences (Table 2). With 100% coverage, 4 sequences could only be matched with sequence similarities lower than 98.7% and may therefore represent new species belonging to the genera *Methanosaeta* (OTU10 and OTU16) or the *Thermoplasmatales*, *Cluster B* (OTU20). The most similar sequences in the databases were from various types of soil environments, water environments and anaerobic bioreactors in North America, Europe and Asia. For 30 of the 82 sequences, the best match came from an anaerobic bioreactor.

**Table 2:** Database comparisons

	**Database match**^**a**^				
**OTU**	**Matching clones**	**Acc. no.**	**Identity**^**b**^	**Taxonomy**	**Source environment**
OTU1	1	CU917405	99.8	*Methanosaeta*	Digester
	6	CU917423	99.6-100	*Methanosaeta*	Digester
	6	CU917466	99.8-100	*Methanosaeta*	Digester
	2	JF280185	100.0	*Methanosaeta*	High mountain hot spring
OTU2	2	AJ831102	99.9	*Archaea*	Landfill drainage layer
	4	CP002565	100.0	*Methanosaeta concilii*	Strain GP6
	1	CU916678	100.0	*Methanosaeta*	Digester
	3	CU917245	99.9-100	*Methanosaeta*	Digester
	2	FR832406	99.9-100	*Methanosaeta concilii*	Digester
OTU3	6	CP002565	99.9-100	*Methanosaeta concilii*	Strain GP6
	1	CU915936	100.0	*Methanosaeta*	Digester
	1	CU916215	99.9	*Methanosaeta*	Digester
OTU4	3	AF050611	99.6-99.9	*Methanosaeta*	Contaminated aquifer
	3	EU155906	99.3	*Archaea*	Rich minerotrophic fen
OTU5	2	AJ831108	99.9	*Archaea*	Landfill drainage layer
	3	CP002565	99.6-100	*Methanosaeta concilii*	Strain GP6
OTU6	4	EU155906	99.0-99.2	*Archaea*	Rich minerotrophic fen
OTU7	4	GU591511	98.8-99.1	*Archaea*	Microbial fuel cell
OTU8	4	GU591511	98.6-99.1	*Archaea*	Microbial fuel cell
OTU9	3	EU155906	98.7-99.2	*Archaea*	Rich minerotrophic fen
	1	AY667272	98.7	*Archaea*	TCE-dechlorinating groundwater
OTU10	1	EU155954	93.5	*Archaea*	Rich minerotrophic fen
	1	FN691755	93.0	*Archaea*	Lake Llebreta
OTU11	1	CU917466	99.9	*Methanosaeta*	Digester
	1	CU916809	99.8	*Methanosaeta*	Digester
OTU12	2	AJ576227	99.5-99.9	*Archaea*	Landfill leachate
OTU13	1	HM244086	99.0	*Archaea*	Lake sediment
	1	AF050611	100.0	*Methanosaeta*	Contaminated aquifer
OTU14	1	HQ592619	99.5	*Archaea*	Activated sludge
OTU15	1	FR749947	98.9	*Methanocorpusculum sinense*	Strain DSM 4274 T
OTU16	1	AY693812	97.6	*Euryarchaea*	Anaerobic sludge
OTU17	1	FR832415	99.8	*Methanosaeta concilii*	Digester
OTU18	1	CU917031	100.0	*Archaea*	Digester
OTU19	1	AJ576235	99.8	*Archaea*	Landfill leachate
OTU20	1	AF050619	98.4	*Euryarchaeota*	Contaminated aquifer
OTU21	1	AB353220	99.2	*Euryarchaeota*	Thermophilic digested sludge
OTU22	1	HQ316970	100.0	*Crenarchaeota*	Wastewater treatment plant, oil refinery
OTU23	1	FR832415	98.8	*Methanosaeta concilii*	Digester
OTU24	1	EU399655	99.2	*Archaea*	Phenol-degrading sludge
OTU25	1	CU917014	99.9	*Archaea*	Digester

^a^ Best matching entry in GenBank or the SILVA rRNA database with 100% coverage. ^b^ Identity in %.

### Phylogenetic tree analysis

**Figure 4:**
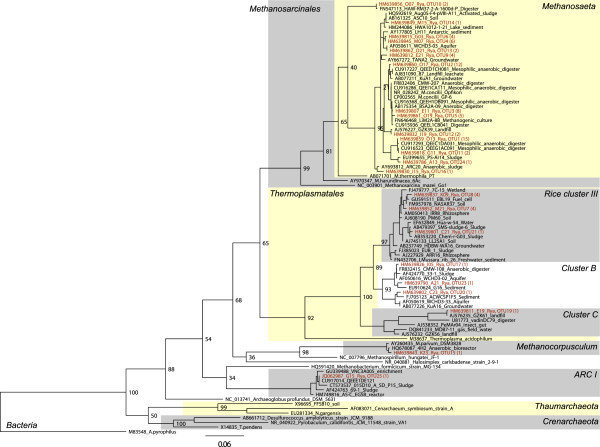
**Phylogenetic tree of archaeal 16S rRNA genes.** Consensus tree constructed from 100 maximum likelihood trees. The branch lengths and the scale bar are proportional to nucleotide differences. Bootstrap values out of a total of 100 are given at the nodes. The sequence of *Aquifex pyrophilus* was used as outgroup. The OTU numbers of the Rya WWTP sequences are given with the total number of sequences within that OTU in parentheses. The cluster names are in accordance with Kemnitz [27], Grosskopf [28] and Chouari [29].

**Figure 5:**
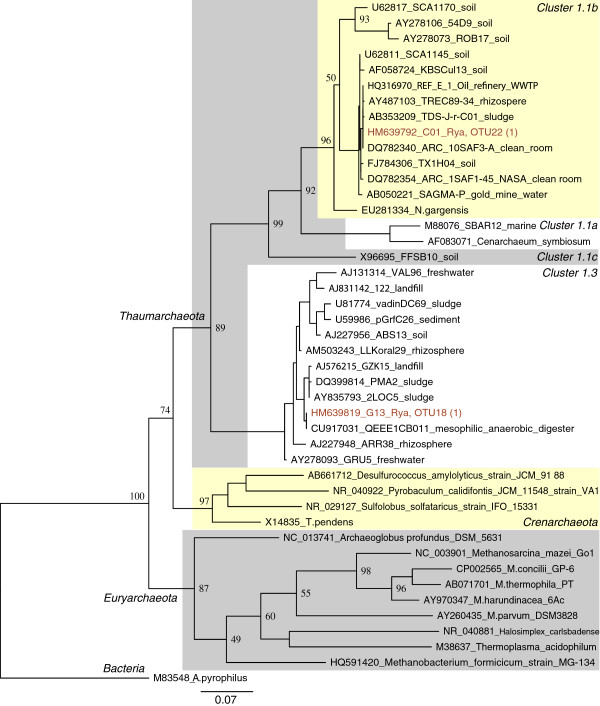
**Phylogenetic tree of archaeal 16S rRNA genes.** Consensus tree constructed from 100 maximum likelihood trees. The branch lengths and the scale bar are proportional to nucleotide differences. Bootstrap values out of a total of 100 are given at the nodes. The sequence of *Aquifex pyrophilus* was used as outgroup. The OTU numbers of the Rya WWTP sequences are given with the total number of sequences within that OTU in parentheses. The cluster names are in accordance with DeLong [30] and Jurgens [31].

The phylogenetic affiliation of the obtained 16S rRNA gene sequences was determined by phylogenetic tree analysis. A phylogenetic tree for *Euryarchaea* inferred by maximum likelihood analysis is shown in Figure 4. A phylogenetic tree for *Crenarchaea* and *Thaumarchaea* inferred by maximum likelihood analysis is shown in Figure 5. The majority of the sequences were determined to be of genus *Methanosaeta* (Figure 3). Several sequences also affiliated with divisions of uncultured *Archaea*.

### Dynamics of the *Archaea* community

**Figure 6:**
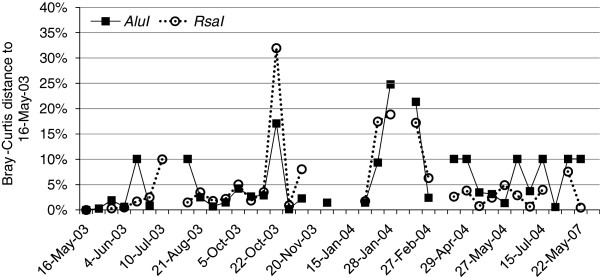
**Community stability.** The stability of the archaeal community is illustrated by plotting the difference, measured as Bray-Curtis distance, between the TRF profile of the first sample in the time series, May 16, 2003, and all following samples. The Bray-Curtis distance is calculated by comparing the relative abundances of the TRFs in the TRF profiles and is low for similar profiles. Results from both the *AluI* and the *RsaI* analysis are shown.

**Figure 7:**
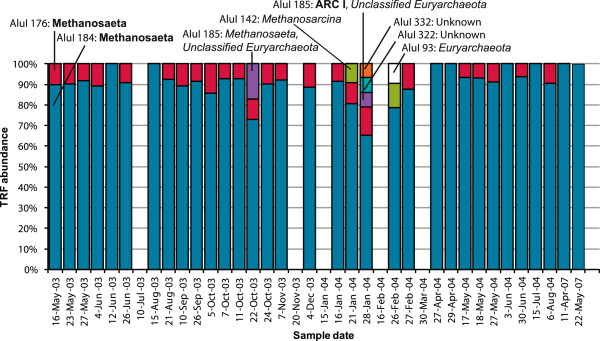
**Relative abundances of *AluI *TRFs.** Relative abundances of TRFs in normalized TRF profiles generated by digestion with *AluI*. Together with the *RsaI* TRFs, the *AluI * TRFs were compared with the predicted TRFs of the clone library sequences (identities in bold) and the sequences from the RDP database (identities in italics) (Table 4).

**Figure 8:**
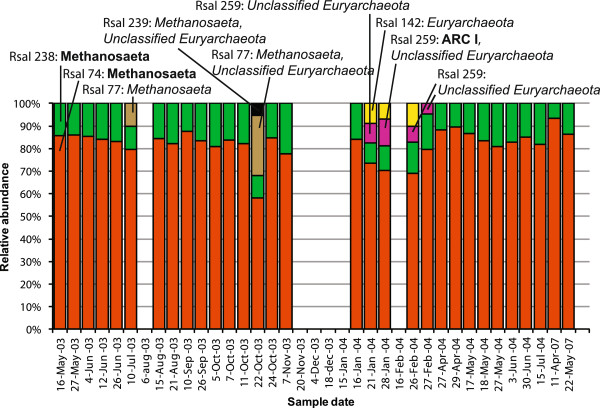
**Relative abundances of *RsaI *TRFs.** Relative abundances of TRFs in normalized TRF profiles generated by digestion with *RsaI*. Together with the *AluI* TRFs, the *RsaI* TRFs were compared with the predicted TRFs of the clone library sequences (identities in bold) and the sequences from the RDP database (identities in italics) (Table 4).

The community composition, as assessed by T-RFLP, changed little throughout the monitored period. The difference, measured as Bray-Curtis distance, between the terminal restriction fragment (TRF) profile of the first sample in the time series, May 16, 2003, and all following samples was on average only 5.8 ± 6.3% and 5.4 ± 7.1% for the *AluI* and *RsaI* analysis, respectively (Figure 6). On two occasions, in October 2003 and in January 2004, the Bray-Curtis distance peaked, indicating a deviation from the community composition at the beginning of the time series. The difference between the TRF profiles of May 16, 2003 and May 22, 2007, four years later, was 10% and 0% for the *AluI* and *RsaI* analysis, respectively. However, the sensitivity of the T-RFLP analysis is limited and TRFs of low abundance cannot be detected. Thus, a Bray-Curtis distance of 0% between the TRF profiles of two samples does not indicate identical community composition since there may be differences in the composition of *Archaea* of low abundance. To get a rough estimate of the sensitivity of the T-RFLP method, a comparison was made between the theoretical TRF profile of the clone library and the observed TRF profile from the same sample. The comparison showed that only TRFs with a relative abundance higher than 20% in the clone library were observed in the TRF profile (Table 3). Thus, the T-RFLP analysis shows the dynamics of the major groups of *Archaea* in the activated sludge. The relative abundances of the observed TRFs in all TRF profiles in the time series are shown in Figures 7 and 8.

**Table 3:** Observed and predicted TRF lengths ^a^

**Observed TRF lengths T-RFLP 2007-05-22**				**Predicted TRF lengths, clone library 2007-05-22**			
***AluI***	**%**^**b**^	***RsaI***	**%**^**b**^	***AluI***	***RsaI***	**%**^**c**^	**Identity**
				84	80	1%	*Thaumarchaeota/Cluster 1.3*
				126	608	1%	*Thermoplasmatales/Cluster B*
				166	609	2%	*Thermoplasmatales/Cluster B*
				180	80	4%	*Methanosaeta*
183	100%	74	86%	188	80	48%	*Methanosaeta*
		238	14%	188	242	21%	*Methanosaeta*
				188	ND^d^	2%	*Methanosaeta*
				189	264	1%	*ARC I*
				359	262	1%	*Thaumarchaeota/Cluster 1.1b*
				400	609	1%	*Thermoplasmatales/RCIII*
				515	264	1%	*Methanocorpusculum*
				551	609	10%	*Thermoplasmatales/RCIII*
				ND	80	1%	*Methanosaeta*
				ND	613	1%	*Thermoplasmatales/Cluster C*
				ND	ND	4%	*Methanosaeta*

^a^ Observed and predicted TRF lengths from T-RFLP and clone library analysis of a sample from 2007-05-22. ^b^ Relative abundance based on total fluorescence. ^c^ Relative abundance based on frequency in clone library. ^d^ ND indicates cases where a TRF could not be predicted or where the predicted TRF was outside the detection range.

To identify the TRFs the observed TRF lengths were compared with the predicted TRF lengths of sequences in the clone library. The predicted TRFs from the sequences in the clone library were between 4 and 6 bases longer than the observed TRFs (Table 3). Such a discrepancy between observed and predicted TRF sizes is commonly observed [32,33]. Not all observed TRFs in the time series could be matched with the predicted TRFs of the clone library sequences. To explore the possibility that the TRFs in the TRF profiles from the samples from 2003 and 2004 come from sequences other than those found in the clone library a comparison was also made with a database of 5802 archaeal 16S rRNA gene sequences matching the primers used in this study. The database was checked for sequences that would result in any of the observed combinations of TRFs generated by *AluI* and *RsaI*. The result of the analysis was a number of possible identities for each observed combination of *AluI* and *RsaI* TRFs (Table 4). Although the database comparison may result in false identities of the TRFs, it is valuable because it gives an indication about the range of species that could give the observed TRF combinations. By comparison with the clone library sequences the dominating TRFs (AluI 176, AluI 184, RsaI 74 and RsaI 238) were determined to represent *Methanosaeta*-like species. Comparisons with the predicted TRFs of 5802 *Archaea* sequences in the database (Table 4) showed that it is possible that the dominating TRFs are from other species of the *Euryarchaeota* than *Methanosaeta.* However, it is unlikely that the origin of these TRFs is species of the *Crenarchaeota* or *Thaumarchaeota*, since all the *Crenarchaeota* or *Thaumarchaeota* sequences in the databases have other predicted TRF lengths.

**Table 4:** Identification of observed TRF combinations

***AluI***^***a***^	***RsaI***^***a***^	**Clone library**^**b**^	**RDP database**^**c**^
93	74	-	Unclassified *Euryarchaota*
142	Out of range^d^	-	*Methanosarcina*
176	74	*Methanosaeta*	*Methanosaeta*
176	238/239	-	*Methanomicrobia*
176	Out of range	-	Unclassified *Euryarchaota*
184/185	74/77	*Methanosaeta*	*Methanosaeta*
			Unclassified *Euryarchaota*
			*Thermoplasmatales*
			*Methanomicrobiales*
			*Methanosarcinales*
184/185	142	-	Unclassified *Euryarchaota*
184/185	238/239	*Methanosaeta*	*Methanosaeta*
			Unclassified *Euryarchaota*
			*Methanomicrobia*
			*Methanosarcinales*
184/185	259	*ARC I*	Unclassified *Euryarchaota*
			*Thermoplasmatales*
			*Methanomicrobiales*
184/185	Out of range	-	Unclassified *Euryarchaota*
			Unclassified *Archaea*
			*Methanosarcinales*
Out of range	74/77	-	Unclassified *Euryarchaota*
			Unclassified *Archaea*
Out of range	238/239	-	Unclassified *Euryarchaota*
			*Methanosarcinales*
Out of range	259	-	Unclassified *Euryarchaota*

^a^ Observed TRF combinations that are not included in the table were not found in the database nor in the clone library. ^b^ Identification by comparison with predicted TRF lengths of clone library sequences. ^c^ Identification by comparison with predicted TRF lengths of RDP database sequences. ^d^ Out of range: The TRF in the database or the clone library was either shorter than 50 bases or longer than 1020 bases and would therefore not have been detected.

### Correlation analysis

Several TRFs showed a significant correlation with process parameters (Table 5). The parameters that correlated with most TRFs were water temperature and nitrogen concentration. There were also significant correlations between several TRFs and the sludge and effluent water properties (Table 6). The parameter effluent non-settleable solids (NSS) and the concentration of carbohydrates in extracted extracellular polymeric substances (EPS) correlated with most TRFs. No TRF showed a significant correlation with the sludge volume or shear sensitivity.

**Table 5:** **Correlations between TRF abundances and WWTP process parameters **^**a**^

***AluI***	**Identity**^**b, c**^	**Observations**^**d**^	**Temp.**^**e**^	**SRT**^**f**^	**F/M**^**g**^	**COD**^**h**^	**NO23-N**^**i**^
AluI 142	*Methanosarcina*^b^	2	**				
AluI 176	*Methanosaeta*^c^	24			**	*	
AluI 184	*Methanosaeta*^c^	33	*		*		
***RsaI***							
RsaI 142	*Euryarchaeota*^b^	3	***				*
RsaI 238	*Methanosaeta*^c^	31	*	*			*
RsaI 259	*ARC I*^c^	4	***				*

^a^ The correlations are marked with asterisks corresponding to the level of statistical significance: 95% (*), 99% (**) and 99.9% (***). TRFs that are not included did not show any statistically significant correlation with any parameter. The WWTP process parameter data was taken from [22]. ^b^ Identification by comparison with the RDP database. ^c^ Identification by comparison with the clone library. ^d^ The number of times the TRF was observed. ^e^ Water temperature (°C). ^f^ Solids retention time (days). ^g^ Food to mass ratio ( g/kg*s). ^h^ Total COD going into the activated sludge tanks (g/s). ^i^ Nitrite/nitrate levels going in to the activated sludge tanks (g/s).

**Table 6:** **Correlations between TRF abundances and sludge and effluent water parameters **^**a**^

***AluI***	**Identity**^**b, c**^	**Observations**^**d**^	**SSVI**^**e**^	**Shear sensitivity**^**f**^	**EPS protein**^**g**^	**EPS carb.**^**h**^	**Effluent NSS**^**i**^
AluI 142	*Methanosarcina*^*b*^	2					***
AluI 176	*Methanosaeta*^*c*^	24					
AluI 184	*Methanosaeta*^*c*^	33				***	***
AluI 185	*ARC I*^*c*^	2			*	***	
***RsaI***							
RsaI 74	*Methanosaeta*^*c*^	31			*	***	
RsaI 142	*Euryarchaeota*^*b*^	3			**	***	***
RsaI 238	*Methanosaeta*^*c*^	31					***
RsaI 259	*ARC I*^*c*^	4			**	***	***

^a^ The correlations are marked with asterisks corresponding to the level of statistical significance: 95% (*), 99% (**) and 99.9% (***). TRFs that are not included did not show any statistically significant correlation with any parameter. The sludge and effluent water parameter data was taken from [22]. ^b^ Identification by comparison with the RDP database. ^c^ Identification by comparison with the clone library. ^d^ The number of times the TRF was observed. ^e^ Standardized sludge volume index (ml/g). ^f^ Shear sensitivity (arbitrary units). ^g^ EPS protein (mg/gMLSS). ^h^ EPS carbohydrates (mg/gMLSS). ^i^ Effluent non-settleable solids (mg/l).

### Quantification and localization of *Archaea* in the activated sludge flocs

**Figure 9:**
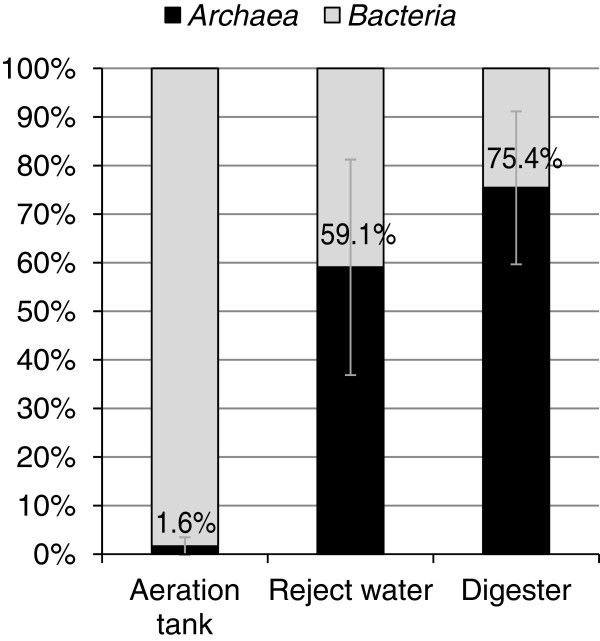
**Quantification of *Archaea *.** Confocal images were collected from triplicate samples from the aeration tank, reject water and the digester. A threshold of 100 was applied to remove noise and *Archaea* and *Bacteria* was quantified as the area positive for ARC915 or MX825 (but not EUB) and EUB (but not ARC915 or MX825), respectively. The given values are average percentages of *Archaea* of the total area with values from 90 confocal images. The standard deviations are given as error bars.

**Figure 10:**
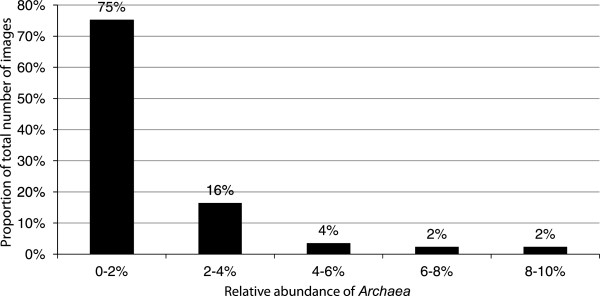
**Distribution of *Archaea *.** The proportion of the total number of confocal images for different intervals of *Archaea* abundance in triplicate samples from the aeration tank.

**Figure 11:**
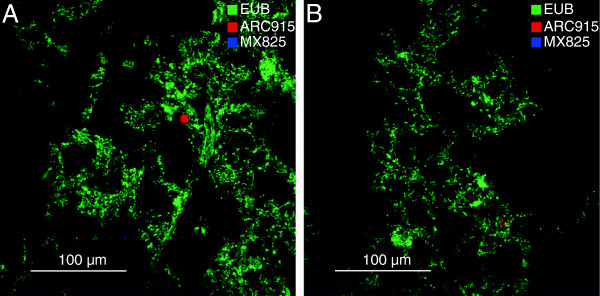
**FISH images with probes for *Bacteria*, *Archaea * and *Methanosaeta*.** Confocal images of activated sludge samples hybridized with probes EUB, ARC915 and MX825, which are specific for *Bacteria*, *Archaea* and *Methanosaeta*, respectively. The images were generated using Daime 1.1 [34] with an applied threshold of 50.

The 16S rRNA gene clone library indicated that published FISH probes would cover the *Archaea* at Rya WWTP. *Archaea* could be observed in the activated sludge flocs, both centrally located and close to the edges of the flocs. FISH analyses showed that the average relative abundance of *Archaea* in the activated sludge of the aeration tank was 1.6% (Figure 9). In the anaerobic digester and in the water recycled into the activated sludge tanks (reject water) there were more *Archaea* than *Bacteria* (Figure 9). In most images of activated sludge flocs the percentage of *Archaea* was lower than 2% (Figure 10). Occasionally there were larger colonies of *Archaea* (Figure 11, panel A) but in most images *Archaea* were either present as individual cells or small colonies (Figure 11, panel B).

## Discussion

In this study the abundance, localization, composition and dynamics of *Archaea* in the activated sludge of a full-scale WWTP were assessed using FISH analysis, 16S rRNA gene clone library analysis and T-RFLP time series analysis. These three analyses were all done on samples collected at different times. However, for most process parameters there were no significant differences between these times (Table 1). The WWTP was also operated the same way at all times, except for four months, May 24 to September 24, 2004, when the primary settlers were bypassed. The samples were therefore considered comparable.

The T-RFLP time series analysis showed that the most abundant TRFs were the same throughout 2003 and 2004 as well as in May 2007 (Figures 7 and 8). If we assume that the same TRF always represent the same group of *Archaea,* then the T-RFLP data show that the main part of the *Archaea* community was the same in 2003, 2004 and in May 2007 (Figures 7 and 8) and that we can use the clone library data to identify the TRFs in the T-RFLP time series. We further assume that the *Archaea* community stayed mainly the same in December 2007, which make it possible to use the clone library data to choose appropriate probes for the FISH analysis.

The clone library sequences indicated that already published FISH probes were relevant for an estimation of the relative abundance of major *Archaea* groups. The relative abundance of the *Archaea* has been estimated in other investigations to be low, based on activity measurements [11], and up to 8% of *Bacteria*[10] or 10% of total cell numbers [16]. In this study *Archaea* was estimated, by FISH, to make up 1.6% of total cell numbers in the activated sludge, a relatively low abundance. However, the importance of a microbial group cannot be deduced by abundance alone. Putative AOA were 1-10% of total cell numbers in activated sludge, but despite this abundance they did not contribute significantly to nitrification [16], whereas foaming organisms have great impact on floc structure and sludge properties even when present in numbers around 1% [35,36]*.* Another example is ammonium oxidizers, which at an abundance of 3-5% (of total bacteria), could perform the first step in a successful 80% reduction of nitrogen in an activated sludge system [37]. Thus, despite their relatively low abundance, a possible contribution of *Archaea* to sludge properties cannot be ruled out.

The composition of the *Archaea* community was investigated by analysis of 82 16S rRNA sequences. The community richness was estimated to be 43 species of 19 genera. As expected, the clone library does not fully cover the *Archaea* community (Figure 1). However, the 25 species of 10 genera that were observed are assumed to represent the most abundant groups. The *Archaea* community was functionally specialized (Figure 2) with 77% of the sequences in the clone library being *Methanosaeta*-like organisms (Figure 3)*.* Specialized communities dominated by methanogens, although of other genera than *Methanosaeta*, have also been observed in activated sludge from other WWTPs [11,12].

The T-RFLP time series analysis showed that the *Archaea* community was practically the same in most samples (Figures 7 and 8), despite variations in environmental conditions such as organic loading rate and temperature [22]. Only in a few samples more than two TRFs were observed. However, as shown in Table 3, the sensitivity of the T-RFLP analysis was low, so it is possible that there were changes in the composition of the less abundant groups of *Archaea*. A comparison between the observed TRF lengths and the predicted TRF lengths of the clone library sequences identified the two main TRFs as coming from *Methanosaeta* sequences, given the assumption that all TRFs represent the same groups of *Archaea* in all samples where they are observed, as discussed above. An alternative way of identifying the TRFs would be to compare the observed *AluI* and *RsaI* TRF combinations with the predicted TRF combinations from *Archaea* sequences in the RDP database. A comparison with 5802 *Archaea* 16S rRNA sequences showed that sequences of *Methanosaeta* or other *Euryarchaea* would give the observed *AluI* and *RsaI* TRF combinations, but no *Crenarchaeota* or *Thaumarchaeota* sequences. In the following discussion we therefore assume that the two main TRFs come from methanogens.

Methanogens are anaerobic and the oxygen concentration in activated sludge is high. However, in the deeper parts of activated sludge flocs anoxic microenvironments can exist [38] which may allow growth of anaerobic organisms. In the activated sludge at Rya WWTP, methanogens were observed both deep within the flocs and close to the surface (Figure 11). Although exposed to oxygen, the methanogens at the surface are not necessarily inactive since methanogens have been shown to be able to maintain viability [11] and activity [39] in the presence of oxygen.

To avoid washout from the activated sludge, microorganisms need to be active and have a doubling time shorter than the sludge retention time. Pure cultures of *Methanosaeta concilii* have a temperature optimum at 35-40°C [40] and a doubling time of 4-7 days at 37°C [41]. The low water temperature at Rya WWTP, 10-20°C, does not necessarily prevent activity since *Methanosaeta*-like species have been shown to grow at 9-14°C in bioreactors [42,43] and dominate methanogenic cultures from rice field soil at 15°C [44]. The solids retention time (SRT) at Rya WWTP is typically calculated as 5-7 days and could also allow for growth of *Methanosaeta*-like organisms. In this study, *Methanosaeta*-like TRFs dominated throughout 15 months, and correlation analysis showed that some of the *Methanosaeta*-like TRFs increased in abundance with increasing temperature and increasing SRTs (Table 5), i.e. theoretically more favorable conditions. Both the constant dominance in the TRF profiles and the response to changed environmental conditions would be explained by an active population of *Methanosaeta*-like organisms. The detection of methanogens by FISH analysis also showed the presence of rRNA, which is expressed in active cells. However, high rRNA levels may be maintained despite inactivity. In conclusion, the activity of the *Methanosaeta*-like organisms is an open question.

If the *Methanosaeta*-like species do not grow fast enough to avoid washout, their constant presence requires that they are constantly added to the sludge. Possible sources are the influent wastewater and recycled water from an anaerobic bioreactor. By FISH analysis, *Archaea* was confirmed to be present in high numbers in both the anaerobic bioreactor and in the reject water (Figure 9). Thus the bioreactor might seed the activated sludge with *Archaea***.** This is supported by the fact that a majority of the detected 16S rRNA sequences cluster with sequences from anaerobic sludge (Figure 4). Furthermore, no sequences matched typical methanogens in human fecal matter, such as *Methanobacter smithii* and *Methanosphaera stadtmanae*[45], indicating that fecal matter from the influent water was not an important input to the methanogens in the activated sludge.

The second largest group in the clone library was *Thermoplasmatales*-related sequences affiliated with *Rice Cluster III* (*RC-III*). No cultured representative of *RC-III Archaea* exists, but a study of a methanogenic enrichment culture suggests that *RC-III Archaea* are mesophilic anaerobes growing heterotrophically on peptides with a doubling time of approximately three days [27]. *RC-III* has been detected in soil [27], anaerobic bioreactors [46] and groundwater [47]. This study shows that *RC-III Archaea* can also be present in activated sludge.

*Thermoplasmatales*-related sequences of *Cluster B* and *C* were also found in the clone library. There are currently no cultured representatives or proposed phenotypes for these groups. *Cluster B* and *C* sequences have been retrieved from environments with methanogenic communities and complete or partially anoxic zones, such as water [48], landfill leachates [49], sediments [50], bioreactors [51] and the digestive tract of animals [52]. This study adds activated sludge to that list.

One sequence, clone G15, belongs to a yet undescribed lineage of *Archaea*: *ARC I*[29]. The *ARC I* lineage is well-represented in anaerobic bioreactors and in reactors with a high abundance of *ARC I*, the abundance of species related to *M. concilii* is low and vice versa [53], which could indicate a competition for acetate between these two lineages. The clone library in this study followed the same pattern with low abundance of ARC I and high abundance of *M. concilii*. The same pattern was also seen in the TRF profiles since the only time that the TRFs corresponding to sequence G15 was observed (January 28, 2004) the relative abundance of the TRFs associated with *M. concilii* had decreased to around 80%.

Archaeal ammonia monooxygenase subunit A (amoA) genes have been observed in various activated sludge WWTPs [13,15]. One of our sequences affiliated with *Crenarchaea* cluster 1.1b, which includes several putative AOA [54-56]. However, it has recently been shown that not all amoA-carrying *Thaumarchaeota* are ammonia-oxidizing autotrophs [57]. The presence of AOA at the Rya WWTP can therefore not be confirmed, and as has been suggested for other WWTPs [14,16], AOA are most likely of minor or no importance for ammonia-oxidation at the Rya WWTP.

One clone affiliated with *Crenarchaea* cluster 1.3. There are no cultured representatives of cluster 1.3, but spatial co-localization [58] and a relation between the abundance of cluster 1.3 and *Methanosaeta*-like species has been reported [42].

In other aggregate structures, such as anaerobic sludge granules, *Methanosaeta* are important for structure and stability and they form dense aggregates which act as nuclei for granule formation [20]. In the activated sludge the *Methanosaeta* did not appear to have this function as they were mostly detected as small colonies or single cells (Figure 11) and there was no apparent difference in structure between flocs with high and low numbers of *Methanosaeta*.

The lowest relative abundances of the *Methanosaeta*-like TRFs were observed in January and February 2004 (Figures 7 and 8). In October 2003 the two main *Methanosaeta* TRFs also decreased in relative abundance but it cannot be ruled out that the TRFs that appeared in those samples were also *Methanosaeta* (Table 4). The lowest water temperatures of the period were recorded during January and February 2004, which could have reduced the survival or proliferation of *Methanosaeta*-like species and allowed other *Archaea* to increase. In anaerobic sludge, a decrease in *Methanosaeta* abundance has been linked to granule disintegration [18,19]. Although the flocs had high shear sensitivity and a more open structure in January and February 2004 when the *Methanosaeta* TRFs decreased and although there was a significant negative correlation between *Methanosaeta* TRFs and effluent non-settleable solids (Table 6) it cannot be concluded that the *Archaea* are important for the floc structure. The increased shear sensitivity and changed floc structure in January and February 2004 could be due to the reduced general microbial activity, which has been shown to decrease floc stability [5]. Furthermore, increased shear sensitivity and changed floc structure was also observed from June to August 2004, after the primary settlers were bypassed, but during this period the relative abundance of the *Methanosaeta* TRFs was 100%. Thus, if the composition of the *Archaea* community has any effect on floc structure or stability it is certainly only one of many other factors.

## Conclusions

By sequencing and T-RFLP analysis of 16S rRNA genes and FISH we showed that *Archaea* were present in the activated sludge of a full-scale WWTP. The *Archaea* community was dominated by *Methanosaeta*-like species, which probably entered the sludge with a stream of recycled water from an anaerobic bioreactor. The *Archaea* were present both as colonies and single cells but only in low numbers, estimated as 1.6% of total cell numbers in the activated sludge. During 15 months major changes in community composition were observed twice, but in both cases the community returned to the previous composition. Even in samples collected three years apart the main part of the community remained the same according to T-RFLP data. We now know that *Archaea* can constitute a small but constant and integral part of the activated sludge and that it can therefore be useful to include *Archaea* in future studies of sludge or floc properties.

## Methods

### Sample collection

The Rya WWTP in Göteborg, Sweden, treats domestic and industrial wastewater serving approximately 850,000 population equivalents. The plant uses pre-denitrification in an activated sludge system and post-nitrifying trickling filters for biological nitrogen removal. Typical sludge age is 5-7 days. A detailed description of the design and operating parameters of the Rya WWTP can be found elsewhere [21]. Samples were collected at the end of the aerated basins. 50 mL of sample was centrifuged and the resulting pellet was stored at -20°C within 1.5 h from collection. For the T-RFLP time series sludge samples were collected between May 16, 2003 and August 6, 2004. The frequency of sample collection varied between days and weeks. One sample was collected May 22, 2007 for T-RFLP and clone library analysis and an additional sample was collected December 12, 2007 for FISH analysis. At all sample times the treatment plant was operated the same way except for four months, May 24 to September 24, 2004, when the primary settlers were bypassed. Table 1 shows average values for some process and sludge parameters during 2003, 2004 and 2007. The software PAST (version 2.01) [59] was used for statistical analysis. The data was not normally distributed and analysis of variance was therefore carried out using the non-parametric Kruskal-Wallis test.

### DNA extraction

DNA was extracted using Power Soil DNA Extraction Kit (MoBio Laboratories). The frozen sludge pellets were thawed, 15 mL sterile water was added and the samples were homogenized by 6 min of mixing in a BagMixer 100 MiniMix (Interscience). Water was removed by centrifugation and DNA was extracted from 0.25 g of homogenized sludge pellet according to the manufacturer’s instructions.

### PCR

Archaeal 16S rRNA genes were amplified using HotStar*Taq*Plus PCR kit (Qiagen) and *Archaea*-specific primers Arch18F (TTCCGGTTGATCCYGCC) and Arch959R (YCCGGCGTTGAMTCCAAT) (Thermo Fisher Scientific). PCR reactions were carried out in a total volume of 20 μl in the provided PCR buffer with 0.5 U HotStarTaq Plus, 200 μM dNTP mix, 0.1 μM of each primer and 2-5 ng DNA. The primers were based on previously published sequences Arch958R and Arch21F [60]. The reverse primer Arch958R was shortened to remove a 4-bases self complementary 3’end (AATT). The forward primer Arch21F was shortened to match the new annealing temperature of the reverse primer. The cycle profiles had an initial 5 min at 95°C for *Taq* polymerase activation followed by denaturation at 94°C for 1 min, annealing at 58°C for 30 s and elongation at 72°C for 1 min. The annealing temperature was decreased 1°C every 3 cycles until reaching 55°C where the number of cycles was 30. The reactions were ended with a final elongation step at 72°C for 7 min.

### Cloning

The PCR-products of nine PCR replicates, generated from two DNA extraction replicates, were pooled and purified using Qiagen MinElute PCR Purification Kit (Qiagen). 8 ng and 15 ng of purified PCR-product were ligated into the plasmid vector pCR 4 TOPO (Invitrogen) in duplicate reactions. One Shot DH5alpha-T1R competent *Escherichia coli* cells (Invitrogen) were transformed with the vector constructs according to the manufacturer’s instructions in two separate reactions. The transformed cells were plated on LB-agar plates with 50 μg/ml Kanamycin and incubated at 37°C over night. 95 cloned sequences were amplified directly from transformed single colonies from the two cloning reactions by PCR using the vector specific primers T3 (ATTAACCCTCACTAAAGGGA) and T7 (TAATACGACTCACTATAGGG). The bacterial cells were lysed by five minutes incubation at 94°C followed by PCR-cycles as described above but with a starting annealing temperature of 57°C.

### Sequencing and sequence analysis

Cloned sequences were sequenced from both ends using Big Dye Sequencing Kit (Applied Biosystems) and primers T3 and T7 as sequencing primers. Sequence data was generated by capillary gel electrophoresis (3730 DNA analyzer, Applied Biosystems). Raw data sequences were manually inspected using SeqScape (Applied Biosystems). Sequences sequenced from different ends of the PCR-product were aligned using BioEdit (version 5.0.9) [61]. Consensus sequences were generated for 82 clones with overlaps between the 5’ and 3’ end sequences ranging from 80 to 496 bases.

The sequences were aligned using the alignment tool of the SILVA rRNA database [26] and checked for chimeras using the Bellerophon server [62]. The sequences were also aligned with a reference E. Coli sequence, accession number U00096, and checked for chimeras using Mallard [63]. No chimeric sequences were detected with either of the two methods.

The similarity between the 16S rRNA gene sequences was determined by generating a similarity matrix using the DNADIST program in the PHYLIP package [64]. The sequences were then assigned to OTUs based on different similarity thresholds. For *Bacteria*, 16S rRNA gene sequence similarities of 80%, 90%, 95% and 98.7% approximately represent the division in phylum, family/class, genus and species levels, respectively [23,24], and we use the same criteria for *Archaea*. Accumulation curves were constructed by plotting the number of OTUs found in a given number of clones versus the number of clones. The lower bound of the richness of the community was estimated with the nonparametric estimator CHAO1 using the software SPADE (version 3.1; Institute of Statistics, National Tsing Hua University http://chao.stat.nthu.edu.tw). The CHAO1 estimator was chosen according to the properties of the data set following the recommendations in the SPADE documentation.

A Pareto-Lorenz evenness curve [65,66] was used to illustrate and quantify the evenness of the *Archaea* community. The sequences were divided in OTUs based on a sequence similarity threshold of 98.7% and ranked from high to low, based on their abundance. The cumulative proportion of OTU abundances (Y) was then plotted against the cumulative proportion of OTUs (X) resulting in a concave curve starting at (X, Y) = (0%, 0%) and ending in (X, Y) = (100%, 100%). The Fo index is the horizontal y-axis projection on the intercept with the vertical 20% x-axis line, i.e. the combined relative abundance of 20% of the OTUs. In a community with high evenness all or most OTUs are equally abundant which results in a Pareto-Lorenz curve close to a straight line of 45^o^. The Fo index for such a community is close to 20%. Specialized communities with one or a few dominating OTUs generate concave curves with high Fo indices.

All sequences were compared with available sequences in the GenBank nucleotide database using BLAST (Basic Local Alignment Search Tool) [25] August 22, 2011. The search tool of the SILVA rRNA database [26] was also used. However, matching sequences in GenBank always had higher similarities than the best matches from SILVA.

TRF lengths were predicted for all clone library sequences. The sequences all started 50-100 bases away from the forward primer so the TRF lengths were predicted by alignment with a reference sequence containing the primer site and assuming that there were no inserts or deletions between the primer and position 100. If the reference sequence had a restriction enzyme cut site preceding the first bases of the clone library sequence, the TRF for the clone library sequence could not be predicted.

25 sequences representing the 25 OTUs obtained by applying a sequence similarity threshold of 98.7% were subjected to phylogenetic analysis. The cloned sequences were aligned together with reference sequences representing known and proposed novel *Archaea* divisions using the alignment tool of the SILVA rRNA database [26]. To make all sequences of equal length the resulting alignment was trimmed using BioEdit [61]. Phylogenetic tree analysis was carried out using the PHYLIP package [64]. Bootstrap analysis was carried out by generating 100 datasets using the program *seqboot*. The 100 datasets were analyzed by the maximum likelihood method using *dnaml* and 100 trees were created. The sequence of the bacteria *Aquifex Pyrophilus* was used as outgroup. A majority rule consensus tree was constructed from the 100 trees using *consense*. The branching of the consensus tree was seen as an estimate of the true tree. Finally, the original alignment was analyzed by maximum likelihood using *dnaml* but instead of searching for the best tree, the sequences were fitted to the consensus tree. In the resulting tree, the branching was derived from the bootstrap analysis, and the branch lengths from the maximum likelihood analysis.

### Nucleotide sequence accession numbers

The partial 16S rRNA gene sequences obtained in this study are available in GenBank under accession numbers JQ062987 and HM639782 to HM639862.

### T-RFLP analysis

Sludge sample collection and DNA extraction was carried out as described above. Archaeal 16S rRNA genes were amplified as described above but with the forward primer Arch18F labeled with the fluorescent dye 6 – carboxyfluorescein. Three PCR reactions were prepared from each sludge sample. The PCR products were purified using the Agencourt AMPure system (Beckman Coulter) and digested with 10 units of restriction enzyme at 37°C for at least 16 hours. Restriction enzymes *AluI* and *RsaI* were used in separate reactions. The restriction digests were purified and analyzed by capillary gel electrophoresis (3730 DNA Analyzer, Applied Biosystems). The size standard LIZ1200 (Applied Biosystems) was used for fragment size determination.

The software Genemapper (Applied Biosystems) was used to quantify the electropherogram data and to generate the TRF profiles. Peaks from fragments of size 50-1020 bases with a height above 50 fluorescent units were analyzed. The total fluorescence of a sample was defined as the sum of the heights of all the peaks in the profile and was interpreted as a measure of the amount of DNA that was loaded on the capillary gel. Only samples with at least two of the three TRF profiles with a total fluorescence above 500 fluorescent units were considered for further analysis. The two profiles with the highest total fluorescence were chosen from each sample. The TRFs of the two profiles were aligned using a moving average procedure [67] and then checked manually for errors. The two profiles were then normalized as described by Dunbar et al [68] and combined to a single consensus profile by taking the average size, height and areas of the fragments present in both. Consensus profiles with a low total fluorescence, i.e. where low amounts of DNA had been loaded on the gel, were excluded from the subsequent analysis to avoid excessive normalization. 32 and 33 consensus TRF profiles, for the *RsaI* and *AluI* analysis, respectively, were normalized and aligned as described above. The TRFs that were removed by normalization constituted only a minor part of the TRF profiles, on average 2 ± 3% and 1 ± 2% of the total fluorescence in the *AluI* and *RsaI* profiles, respectively.

The dynamics of the *Archaea* community were evaluated by pair-wise comparisons of TRF profiles using the Bray-Curtis distance coefficient (described in e.g. [69]). The Bray-Curtis distance coefficient is calculated using the relative abundances of the TRFs. All TRF profiles were compared with the TRF profile of the first sample in the time series.

Possible identities of the TRFs were investigated as follows. The Virtual digest tool at the MICA website [70] was used to generate a list of 5802 sequences from the RDP database (*RDP (R10, U26) 70108 16S rRNA Archaeal*) that matched the primers 18F and 959R. For each sequence the predicted TRF lengths after digestion with *RsaI* and *AluI *were given. Sequences in the list that had both *AluI* and *RsaI* TRFs that matched the TRFs in the observed TRF profiles were selected. The selection was done using a Visual Basic macro for Microsoft Office Excel (Microsoft Corporation) (available from corresponding author). The sequences of the possible candidates were obtained from Genbank and fed into the RDP classifier [71]. Each observed TRF could then be assigned various possible taxonomic classes.

The relative abundance of the TRFs was calculated as the peak height of the TRF divided by the total fluorescence of the TRF profile. The Pearson’s product momentum correlation coefficient was used to estimate the linear correlation between relative abundances of TRFs, process parameters and sludge properties. For details on the process data and sludge properties measurements, see [22]*.* To determine the statistical significance of the correlation a t-test was carried out.

### Fluorescence in situ hybridization

Samples were collected from the anaerobic digester, the reject water and the aeration tank and fixed in 4% paraformaldehyde at 4°C for 3 h. The fixed samples were washed with phosphate-buffered saline (PBS) and stored in PBS-ethanol (1:1) at −20°C until analysis. The hybridization protocol was based on previously published protocols [72]. In short, 3 aliquots of 3 μl fixed sample were applied to microscope slides, air-dried and dehydrated by incubation in ethanol. 30 μl of hybridization buffer containing probe and formamide was applied to each aliquot and in situ hybridization with labeled rRNA-targeted probes was performed in humidity chambers at 46°C for 2 h. The slides were washed with washing buffer, rinsed in ice-cold water and air-dried. To prevent fluorochrome bleaching, all slides were mounted with Citifluor AF1 (Citifluor Ltd, London, UK). Target sequences, hybridization conditions, and references for the probes used in this study are listed in Table 7. All fluorescent probes were obtained from Thermo Hybaid (Interactiva Division, Ulm, Germany). Fluorescent probes were labeled at the 5' end with indocarbocyanine (Cy3), indodicarbocyanine (Cy5) or Alexa Fluor 488.

**Table 7:** FISH probes targeting 16S rRNA and the hybridization conditions used in this study

**Probe**	**Target**	**Target sequence**	***E.coli *positions**	**Formamide (%)**	**NaCl (mM)**	**Fluorophore**	**Reference**
ARC915	*Archaea*	GTGCTCCCCCGCCAATTCCT	915-934	35	70	Cy5	[73], [74]
MX825	*Methanosaetaceae*	TCGCACCGTGGCCGACACCTAGC	825-845	50	18	Cy3	[73], [74]
EUB338	*Bacteria*	GCTGCCTCCCGTAGGAGT	338-355	35	70	Alexa	[75]
EUB338 II	*Bacteria*	GCAGCCACCCGTAGGTGT	338-355	35	70	Alexa	[75]
EUB338 III	*Bacteria*	GCTGCCACCCGTAGGTGT	338-355	35	70	Alexa	[75]

### Quantification using FISH

Confocal images were collected with a Bio-Rad Radiance 2000 MP microscope (Bio-Rad, Hemel Hempstead, UK) using the Ar Kr/Ar (488 nm), GHe/Ne (543 nm) and Red Diode (638 nm) lasers and the bundled software LaserSharp 2000. Settings: 40xOil inverted objective (Nikon Eclipse TE300 Corp, Tokyo, Japan), Image size: 512x512 pixel, XY-pixel: 0.60 μm, Kalman filtration (n = 3). For each sample, three replicates were analyzed. For each replicate, images were collected from 10 fields of view, chosen by arbitrary movements in the X-Y-direction. For each field of view, 3 images were collected at 4 μm intervals in the Z-direction. In total 90 images were collected per sample.

The images were analyzed using ImageJ (version 1.44p, Wayne Rasband, National Institute of Health, Bethesda, MD, USA, available at the public domain at http://rsb.info.nih.gov/ij/index.html). A threshold of 100 was applied to remove noise. Images were converted to binary images and image calculations using the AND and OR functions were applied as follows. Cells stained with both EUBmix and either one of ARC915 or MX825 were removed from further analysis. The combined area of *Archaea* and *Bacteria*-positive cells, the total area with a signal, was calculated for all 90 images and all 3 probes. ARC915, although designed as a universal *Archaea* probe did not cover all MX825 positive cells. The total area for *Archaea* was therefore counted as ARC915 positive cells plus MX825 positive cells not covered by ARC915. The relative abundance of *Archaea* was then calculated as the total area of *Archaea* divided by the combined area of *Archaea* and *Bacteria*. To analyze only images of flocs, and not dispersed cells, images with a total area (both *Bacteria* and *Archaea*) lower than 1000 pixels were removed.

Daime 1.1 [34] was used to generate images with all three probes used in the FISH analysis.

## Authors’ contributions

NJF, MH and BMW conceived and designed the study. NJF and BMW collected samples. NJF carried out the experiments, evaluated the results and drafted the manuscript. BMW and MH provided guidance during the whole study and revised the manuscript. All authors read and approved the final manuscript.
